# Factors Associated With Racial Differences in Deaths Among Nursing Home Residents With COVID-19 Infection in the US

**DOI:** 10.1001/jamanetworkopen.2020.37431

**Published:** 2021-02-10

**Authors:** Rebecca J. Gorges, R. Tamara Konetzka

**Affiliations:** 1 Center for Health and the Social Sciences, University of Chicago, Chicago, Illinois; 2 Department of Public Health Sciences, Biological Sciences Division, University of Chicago, Chicago, Illinois; 3 Department of Medicine, Biological Sciences Division, University of Chicago, Chicago, Illinois

## Abstract

**Importance:**

It is important to understand differences in coronavirus disease 2019 (COVID-19) deaths by nursing home racial composition and the potential reasons for these differences so that limited resources can be distributed equitably.

**Objective:**

To describe differences in the number of COVID-19 deaths by nursing home racial composition and examine the factors associated with these differences.

**Design, Setting, and Participants:**

This cross-sectional study of 13 312 nursing homes in the US used the Nursing Home COVID-19 Public File from the Centers for Medicare and Medicaid Services, which contains COVID-19 cases and deaths among nursing home residents as self-reported by nursing homes beginning between January 1, 2020, and May 24, 2020, and ending on September 13, 2020. Data were analyzed from July 28 to December 18, 2020.

**Exposures:**

Confirmed or suspected COVID-19 infection. Confirmed cases were defined as COVID-19 infection confirmed by a diagnostic laboratory test. Suspected cases were defined as signs and/or symptoms of COVID-19 infection or patient-specific transmission-based precautions for COVID-19 infection.

**Main Outcomes and Measures:**

Deaths associated with COVID-19 among nursing home residents. Death counts were compared by nursing home racial composition, which was measured as the proportion of White residents.

**Results:**

Among 13 312 nursing homes included in the study, the overall mean (SD) age of residents was 79.5 (6.7) years. A total of 51 606 COVID-19–associated deaths among residents were reported, with a mean (SD) of 3.9 (8.0) deaths per facility. The mean (SD) number of deaths in nursing homes with the lowest proportion of White residents (quintile 1) vs nursing homes with the highest proportions of White residents (quintile 5) were 5.6 (9.2) and 1.7 (4.8), respectively. Facilities in quintile 1 experienced a mean (SE) of 3.9 (0.2) more deaths than those in quintile 5, representing a 3.3-fold higher number of deaths in quintile 1 compared with quintile 5. Adjustment for the number of certified beds reduced the mean (SE) difference between these 2 nursing home groups to 2.2 (0.2) deaths. Controlling for case mix measures and other nursing home characteristics did not modify this association. Adjustment for county-level COVID-19 prevalence further reduced the mean (SE) difference to 1.0 (0.2) death.

**Conclusions and Relevance:**

In this study, nursing homes with the highest proportions of non-White residents experienced COVID-19 death counts that were 3.3-fold higher than those of facilities with the highest proportions of White residents. These differences were associated with factors such as larger nursing home size and higher infection burden in counties in which nursing homes with high proportions of non-White residents were located. Focusing limited available resources on facilities with high proportions of non-White residents is needed to support nursing homes during potential future outbreaks.

## Key Points

**Question:**

Are case mix, nursing home characteristics, and community spread of coronavirus disease 2019 (COVID-19) associated with racial differences in deaths among nursing home residents with COVID-19 infection in the US?

**Findings:**

In this cross-sectional study of 13 312 nursing homes in the US, deaths among nursing home residents with COVID-19 infection were higher in facilities that had low proportions (<60%) of White residents compared with those that had high proportions (>97%) of White residents. This difference in mortality was associated with a combination of differences in facility characteristics and location.

**Meaning:**

The study’s findings suggest that the inequalities underlying racial disparities in COVID-19 infection and mortality in the general population may also be associated with differences in mortality among nursing home residents with COVID-19 infection.

## Teaser

This cross-sectional study uses data from the Centers for Medicare and Medicaid Services to describe the racial differences in the number of COVID-19 deaths among nursing home residents by the racial composition of the facility and to examine the factors associated with these differences.

## Introduction

Deaths associated with coronavirus disease 2019 (COVID-19) infection in the US have been disproportionately higher among individuals from racial and ethnic minority groups compared with White individuals.^1^ Given that 40% of COVID-19 deaths have been associated with long-term care facilities,^2^ it is not surprising that the disparities observed among individuals with COVID-19 in the general population are also present among nursing home residents with COVID-19.^3^ In this study, we investigated the differences in COVID-19 cases and deaths by the racial composition of nursing home residents and examined the factors associated with the high rates of COVID-19 deaths found in nursing homes with the largest proportions of residents from racial and ethnic minority groups.

Before the COVID-19 pandemic began, it was well established that racial disparities in the quality of nursing home care were common.^4^ Relative to White individuals, Black individuals are more likely to be admitted to the lowest-quality nursing homes,^5^ which have lower nurse staffing ratios, more serious regulatory deficiencies, and a higher likelihood of being terminated from the Medicaid program.^6,7,8^ Disparities in care appear to be largely a function of where people go for care rather than differential treatment by staff members within the same facilities, as nursing homes are highly segregated.^8^ These disparities have also been associated with payment source. Nursing homes are primarily funded by public payers, with approximately two-thirds of residents in a typical facility covered by Medicaid insurance, and the proportion of residents with Medicaid coverage at the facility level has been associated with between-facility (but not within-facility) differences in quality, broadly defined.^7^

Disparities in COVID-19 deaths may be expected given these differences in nursing home quality, but research to date has revealed little association between nursing home quality ratings and COVID-19 cases and deaths.^9,10,11^ Nursing homes with higher proportions of non-White residents may be especially susceptible to COVID-19 deaths for several additional reasons that are more specific to the nature of the pandemic.^12^ First, non-White residents are more likely to live in facilities that are larger, which creates more opportunity for viral transmission. Second, non-White residents generally enter nursing homes in worse health,^13^ which may be associated with higher COVID-19 mortality. Third, non-White residents are more likely to live in facilities that are for profit, more reliant on Medicaid, and have deficiencies in care.^7,14,15^ Fourth, COVID-19 is more prevalent in non-White communities, and community spread is a factor associated with cases and deaths in nursing homes.^10,11^ Our goal in this study was to investigate the racial factors associated with COVID-19 deaths among nursing home residents by examining the association between the racial composition of nursing home residents and COVID-19 deaths and assessing whether that association was attenuated when facility size, case mix, other facility characteristics, and community prevalence of COVID-19 were taken into account.

## Methods

The study sample consisted of 13 312 nursing homes across the US that reported COVID-19 data to the Centers for Disease Control and Prevention, passed quality assurance reviews, and had compete information available on nursing home characteristics (a full list of inclusion criteria is available in eTable 1 in the Supplement). This study was determined to be exempt from institutional review board review by the University of Chicago Biological Sciences Division owing to the use of publicly available data. The study followed the Strengthening the Reporting of Observational Studies in Epidemiology (STROBE) reporting guideline for cross-sectional studies.

We merged the Nursing Home COVID-19 Public File from the Centers for Medicare and Medicaid Services with COVID-19 cases from the USAFacts website, the LTCfocus^16^ database, and the Nursing Home Compare archives. The Centers for Medicare and Medicaid Services Nursing Home COVID-19 Public File is derived from the COVID-19 long-term care facility module provided by the National Healthcare Safety Network system of the Centers for Disease Control and Prevention. Beginning on May 24, 2020, all nursing homes with Medicaid and Medicare certification have been required to submit weekly reports to the Centers for Disease Control and Prevention via the module. The Centers for Medicare and Medicaid Services then conducts basic quality assurance reviews and releases reports for the nursing homes that pass these reviews on a public website. Nursing homes are permitted to update incorrect data in subsequent weekly reports. We analyzed data that were released on December 4, 2020.

### Definitions of Measures

The primary outcome was facility-level count of COVID-19 deaths among nursing home residents as self-reported by nursing homes beginning between January 1, 2020, and May 24, 2020, and ending on September 13, 2020. We also examined the total number of confirmed and suspected COVID-19 cases among residents over the same period. Confirmed cases were defined as COVID-19 infection confirmed by a diagnostic laboratory test. Suspected cases were defined as signs and/or symptoms of COVID-19 infection or patient-specific transmission-based precautions for COVID-19 infection. We calculated the number of facility-level deaths per 100 cases from these 2 measures.

Nursing home racial composition was measured as the proportion of residents of non-Hispanic White race contained in the LTCfocus database, grouped into 5 racial composition quintiles across the sample. Quintile categories were based on the percentage of nursing home residents who were White (quintile 1 indicated 0% to 59.73%, quintile 2 indicated 59.75% to 80.99%, quintile 3 indicated 81.00% to 91.77%, quintile 4 indicated 91.78% to 97.32%, and quintile 5 indicated 97.33% to 100%). The proportion of White residents was derived from self-reported data provided by nursing home residents as part of Minimum Data Set assessments. The available data did not allow us to examine all non-White groups separately. However, to assess possible differences among groups that we could identify, we used an alternative categorization of nursing homes in supplemental analyses, classifying homes as having high proportions of White, Black, Hispanic or Hispanic and Black residents by using the percentage of residents in each of the 3 race/ethnicity categories available in the LTCfocus database.

Case mix was measured using the LTCfocus acuity index and the proportion of residents with hypertension. The acuity index captures functional limitations and cognitive impairment among a facility’s residents. Acuity index scores are calculated by LTCfocus at the facility level, and scores range from 0 to 27, with 0 indicating no functional or cognitive impairment among any of the residents and 27 indicating severe limitations and the need for several specialized services among all residents. In the nursing homes included in this study, acuity index scores ranged from 0 to 22.5. We controlled for nursing home characteristics that were reported to be associated with COVID-19 deaths in previous studies^10,11^; these characteristics included the number of certified beds (which was used as a proxy for facility size), nursing home ownership (for-profit, government, or nonprofit), chain membership, payer mix (Medicaid and Medicare), Nursing Home Compare overall star rating (range, 1-5 stars, with 1 star indicating lowest quality and 5 stars indicating highest quality), and case mix–adjusted nursing hours (measured as hours per resident per day). Community spread was measured as the number of confirmed COVID-19 cases (excluding cases among nursing home residents) per 1000 people in the county.

### Statistical Analysis

First, we computed unadjusted means to describe the characteristics of nursing homes by racial composition quintile. Next, we used multivariable regression models to estimate the marginal effects of quintile for the number of deaths, sequentially adding adjustments for number of beds, case mix, other facility characteristics, and county-level COVID-19 cases. Because the number of deaths was a count outcome that included a large number of facilities reporting 0 deaths, we used zero-inflated negative binomial models.^17^ We also repeated the main analyses using negative binomial models to determine the extent to which the findings were sensitive to the specific count data model used (eTable 3 in the Supplement). To aid the interpretation of the regression analysis results, we reported average marginal effects estimates and SEs for each quintile relative to the base category of quintile 5, which represented the group of nursing homes with the highest proportion of White residents. We also used stratification, dividing the sample into 5 groups based on the nursing homes’ overall star ratings from the Nursing Home Compare archives to examine whether associations observed in the full sample were different for nursing homes with different ratings.

Stata software, version 15.1 (StataCorp LLC) was used for all analyses. Statistical tests were 2-sided with a significance threshold of *P* < .05. Data were analyzed from July 28 to December 18, 2020.

## Results

Among 13 312 nursing homes included in the study, the mean (SD) age of residents was 79.5 (6.7) years, and 58.6% were female. As of September 13, 2020, 334 844 COVID-19 cases and 51 606 COVID-19–associated deaths were reported among residents, with a mean (SD) of 3.9 (8.0) deaths per facility. A total of 10 494 nursing homes (78.8%) reported at least 1 COVID-19 case, and 5794 nursing homes (43.5%) reported at least 1 COVID-19–associated death. The mean (SD) proportion of White residents was 77.9% (24.3%), ranging from less than 59.7% in quintile 1 facilities to more than 97.3% in quintile 5 facilities (Table 1). The mean (SD) number of deaths in nursing homes with the highest proportions of non-White residents (quintile 1) vs nursing homes with the highest proportions of White residents (quintile 5) were 5.6 (9.2) and 1.7 (4.8), respectively.

**Table 1.** Unadjusted Nursing Home Characteristics by Racial Composition Quintile^a^

Characteristic	Quintile, mean (SD)^b^				
	1 (Low)	2	3	4	5 (High)
Total nursing homes, No.	2589	2669	2691	2698	2665
Certified beds	127.8 (77.4)	121.2 (59.3)	112.5 (58.5)	103.3 (53.9)	87.9 (44.1)
Acuity index^c^	12.6 (1.9)	12.4 (1.3)	12.2 (1.2)	12.1 (1.0)	11.8 (1.1)
Percent of residents with hypertension	76.9 (11.6)	76.6 (10.3)	76.2 (10.4)	76.2 (10.5)	75.7 (10.7)
Ownership, No. (%)					
For profit	2169 (83.8)	2150 (80.6)	2021 (75.1)	1797 (66.6)	1338 (50.2)
Government	129 (5.0)	111 (4.2)	145 (5.4)	168 (6.2)	247 (9.3)
Nonprofit	291 (11.2)	408 (15.3)	525 (19.5)	733 (27.2)	1080 (40.5)
Chain membership, No. (%)	1486 (57.4)	1703 (63.8)	1670 (62.1)	1646 (61.0)	1379 (51.7)
Primary payer					
Percent of residents with Medicaid as primary payer	69.7 (20.8)	64.7 (20.0)	59.2 (21.7)	55.8 (22.4)	53.5 (21.0)
Percent of residents with Medicare as primary payer	11.2 (9.8)	13.7 (11.4)	15.1 (12.9)	14.7 (12.8)	11.9 (10.8)
Overall NHC star rating	2.7 (1.4)	2.8 (1.4)	3.1 (1.4)	3.3 (1.4)	3.6 (1.3)
Adjusted nursing h/resident/d	3.7 (0.8)	3.7 (0.7)	3.8 (0.7)	3.9 (0.8)	4.0 (0.8)
COVID-19 cases					
Nursing homes reporting ≥1 case among residents, No. (%)^d^	2262 (87.4)	2253 (84.4)	2156 (80.1)	2018 (74.8)	1805 (67.7)
Cases per nursing home^d^	37.1 (46.2)	31.5 (42.8)	25.4 (37.6)	19.9 (32.7)	12.3 (24.2)
County-level cases per 1000 people^e^	23.2 (10.7)	20.1 (10.0)	17.3 (10.1)	13.9 (8.4)	12.1 (8.9)
COVID-19 deaths^d^					
Nursing homes reporting ≥1 death among residents, No. (%)	1557 (60.1)	1474 (55.2)	1219 (45.3)	958 (35.5)	586 (22.0)
Deaths per nursing home	5.6 (9.2)	5.2 (9.5)	3.8 (7.4)	3.2 (7.6)	1.7 (4.8)
Facility-level deaths per 100 cases	15.9 (22.6)	15.5 (22.1)	13.2 (20.7)	11.8 (20.6)	8.0 (17.2)

Abbreviations: COVID-19, coronavirus disease 2019; NHC, Nursing Home Compare.

^a^ Data were obtained from the Centers for Medicare and Medicaid Services COVID-19 nursing home data set that was released on December 4, 2020. This information was then merged with data from the LTCfocus database and NHC archives. Analysis was limited to the 13 312 facilities that reported COVID-19 case data, passed Centers for Medicare and Medicaid Services quality assurance reviews, and did not have missing data regarding the percentage of White residents.

^b^ Nursing homes were categorized by racial composition quintile based on the percentage of White residents, with quintile 1 indicating 0% to 59.73%, quintile 2 indicating 59.75% to 80.99%, quintile 3 indicating 81.00% to 91.77%, quintile 4 indicating 91.78% to 97.32%, and quintile 5 indicating 97.33% to 100% White. Differences by racial composition quintile were statistically significant (*P* < .001) for all variables using a linear regression model for continuous measures and a Pearson χ^2^ test for binary and categorical measures.

^c^ Acuity index scores are calculated by LTCfocus on the facility level, and scores range from 0 to 27, with 0 indicating no functional or cognitive impairment among any of the residents and 27 indicating severe limitations and the need for several specialized services among all residents. In the nursing homes included in this study, acuity index scores ranged from 0 to 22.5.

^d^ Cases and deaths comprise the total number of confirmed and suspected COVID-19 cases and deaths among nursing home residents through September 13, 2020.

^e^ County-level cases per 1000 people comprise confirmed cases (excluding cases among nursing home residents) based on data from USAFacts as of September 13, 2020.

Compared with nursing homes with high proportions of non-White residents (quintile 1), nursing homes with high proportions of White residents (quintile 5) were located in counties with fewer confirmed COVID-19 cases per capita (mean [SD], 23.2 [10.7] cases per 1000 people vs 12.1 [8.9] cases per 1000 people), were less likely to be for profit (2169 facilities [83.8%] vs 1338 facilities [50.2%]), and had fewer certified beds (mean [SD], 127.8 [77.4] beds vs 87.9 [44.1] beds), better resident health as measured by the acuity index (mean [SD] score, 12.6 [1.9] vs 11.8 [1.1]) and the percentage of residents with hypertension (mean [SD], 76.9% [11.6%] residents vs 75.7% [10.7%] residents), a lower proportion of residents with Medicaid coverage (mean [SD], 69.7% [20.8%] residents vs 53.5% [21.0%] residents), a higher proportion of residents with Medicare coverage (mean [SD], 11.2% [9.8%] residents vs 11.9% [10.8%] residents), higher star ratings (mean [SD], 2.7 [1.4] stars vs 3.6 [1.3] stars), and higher nursing hours (mean [SD], 3.7 [0.8] hours per resident per day vs 4.0 [0.8] hours per resident per day) (Table 1).

**Figure 1.**
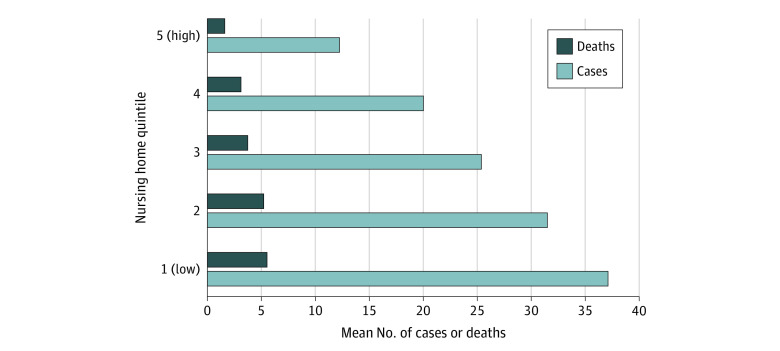
Unadjusted Coronavirus Disease 2019 (COVID-19) Cases and Deaths Among Residents by Nursing Home Racial Composition Quintile A total of 13 312 facilities were included. The analysis was limited to facilities that reported COVID-19 case data, passed quality assurance reviews from the Centers for Medicare and Medicaid Services, and were not missing data regarding the percentage of White residents. Data were obtained from the Centers for Medicare and Medicaid Services COVID-19 nursing home data set that was released on December 4, 2020. This information was then merged with data from the LTCfocus database and Nursing Home Compare archives. Nursing homes were categorized by racial composition quintile based on the percentage of White residents, with quintile 1 indicating 0% to 59.73%, quintile 2 indicating 59.75% to 80.99%, quintile 3 indicating 81.00% to 91.77%, quintile 4 indicating 91.78% to 97.32%, and quintile 5 indicating 97.33% to 100%. Cases and deaths comprise the total number of confirmed and suspected COVID-19 cases and deaths among nursing home residents through September 13, 2020.

The number of COVID-19 cases and deaths decreased monotonically with the percentage of White residents (Figure 1). The mean (SD) number of cases among nursing homes with the highest proportions of White residents (quintile 5) was 12.3 (24.2), while the mean (SD) number of cases among nursing homes with the lowest proportions of White residents (quintile 1) was 37.1 (46.2) (Table 1). Facilities in quintile 1 experienced a mean (SE) of 3.9 (0.2) more deaths than those in quintile 5, representing a 3.3-fold higher number of deaths in quintile 1 compared with the mean (SD) of 1.7 (4.8) deaths among facilities in quintile 5. The mean (SD) number of facility-level deaths per 100 cases was 15.9 (22.6) among facilities in quintile 1, which was 2-fold higher than the mean (SD) number of 8.0 (17.2) deaths per 100 cases among facilities in quintile 5 (Table 1).

**Figure 2.**
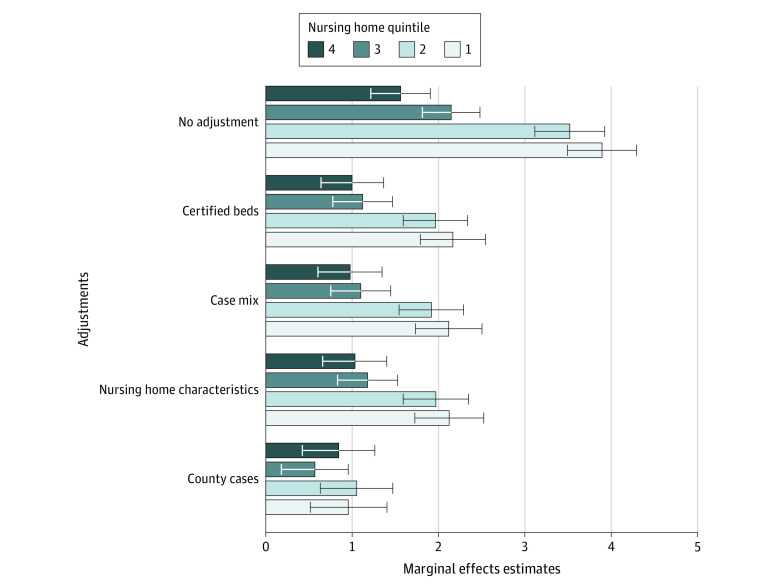
Marginal Effects Estimates by Nursing Home Racial Composition Quintile for Number of Coronavirus Disease 2019 (COVID-19) Deaths Among Residents Marginal effects and 95% CIs were determined using zero-inflated negative binomial regression analysis. Marginal effects were relative to quintile 5, the group of nursing homes with the highest proportion of White residents. Nursing homes were categorized by racial composition quintile based on the percentage of White residents, with quintile 1 indicating 0% to 59.73%, quintile 2 indicating 59.75% to 80.99%, quintile 3 indicating 81.00% to 91.77%, quintile 4 indicating 91.78% to 97.32%, and quintile 5 indicating 97.33% to 100%. The no-adjustment model calculated the differences from quintile 5 by the percentage of quintile in mean death counts. The certified beds model was adjusted for the number of certified beds only. The case mix model was adjusted for the number of certified beds, acuity index, and percentage of residents with hypertension. The nursing home characteristics model was adjusted for the number of certified beds, case mix, and nursing home characteristics (ownership, chain membership, percentage of residents with Medicaid coverage, percentage of residents with Medicare coverage, Nursing Home Compare overall star rating, and adjusted nursing hours per resident per day). The county-level cases model was adjusted for confirmed COVID-19 cases (excluding cases among nursing home residents) per 1000 people per county using the quadratic functional form equation (the sum of x plus x^2^) in addition to the number of certified beds, case mix, and nursing home characteristics. Errors bars represent 95% CIs.

These unadjusted differences in death counts by nursing home racial composition decreased when sequentially adjusting for characteristics that differed across the nursing home quintile groups. After adjustment for the number of certified beds in a facility, nursing homes in quintile 1 were associated with a mean (SE) of 2.2 (0.2) more deaths than facilities in quintile 5 (Figure 2; eTable 2 in the Supplement). Adjusting for case mix and additional nursing home characteristics did not modify this association. An analysis adjusted for county-level COVID-19 cases indicated further reduction in the differences between racial groups; however, even in the fully adjusted model, nursing homes with the highest proportions of non-White residents had a mean (SE) of 1.0 (0.2) additional COVID-19 death compared with nursing homes with the lowest proportions of non-White residents. Nursing homes with more certified beds (marginal effect [SE] of a 1-unit increase in the natural log of the number of certified beds, 4.831 [0.165] deaths), larger proportions of residents with hypertension (marginal effect [SE], 0.015 [0.006]), fewer residents with Medicare coverage (marginal effect [SE], −0.013 [0.007]), and fewer hours of nursing care (marginal effect [SE], −0.549 [0.091]) had more COVID-19–associated deaths (eTable 3 in the Supplement).

These associations were largely consistent within each of the Nursing Home Compare star rating categories in the stratified analyses (Table 2). However, the differences in death counts by racial composition were largest among 1-star (lowest-quality) facilities. For example, among 1-star facilities, nursing homes in quintile 1 had a mean (SE) of 2.3 (0.6) more deaths than those in quintile 5, which represented the largest marginal effects for quintile 1 of any of the star-rating categories. The marginal effects of adjusted nursing hours per resident per day were only statistically significant for nursing homes with 4 or 5 stars and not those with lower star ratings.

**Table 2.** Marginal Effects Estimates by Overall Nursing Home Compare Star Rating^a^

Variable	COVID-19 deaths, marginal effects (SE)				
	1-Star facilities	2-Star facilities	3-Star facilities	4-Star facilities	5-Star facilities
Total nursing homes, No.	2327	2655	2486	2895	2949
Quintile^b^					
1	2.335 (0.651)^c^	0.263 (0.719)	0.838 (0.508)	1.015 (0.399)^d^	0.354 (0.381)
2	1.670 (0.579)^c^	0.714 (0.676)	1.065 (0.482)^d^	0.670 (0.370)	0.988 (0.385)^d^
3	0.999 (0.583)	−0.298 (0.637)	0.552 (0.442)	0.834 (0.363)^d^	0.514 (0.323)
4	0.926 (0.600)	−0.571 (0.665)	0.598 (0.539)	1.286 (0.372)^c^	1.078 (0.334)^c^
ln (certified beds)	5.137 (0.446)^c^	5.676 (0.423)^c^	4.486 (0.395)^c^	4.702 (0.311)^c^	4.113 (0.295)^c^
Case mix index	0.048 (0.111)	−0.029 (0.143)	0.033 (0.099)	0.101 (0.093)	−0.111 (0.104)
Residents with hypertension, %	0.014 (0.017)	0.014 (0.016)	0.013 (0.014)	0.016 (0.011)	0.016 (0.011)
Ownership					
For profit	0.609 (0.590)	0.282 (0.508)	0.458 (0.398)	−0.162 (0.280)	−0.327 (0.249)
Government	0.220 (1.024)	−0.448 (1.045)	0.227 (0.626)	−0.714 (0.605)	−1.468 (0.555)^c^
Chain membership	0.424 (0.304)	−0.459 (0.345)	−0.440 (0.288)	−0.285 (0.240)	−0.143 (0.217)
Primary payer					
Percent of residents with Medicaid as primary payer	−0.035 (0.011)^c^	0.017 (0.009)	−0.008 (0.010)	−0.005 (0.007)	−0.005 (0.006)
Percent of residents with Medicare as primary payer	−0.008 (0.020)	−0.016 (0.020)	−0.016 (0.015)	−0.009 (0.013)	−0.014 (0.010)
Adjusted nursing h/resident/d	0.021 (0.265)	−0.210 (0.255)	−0.148 (0.249)	−0.816 (0.189)^c^	−0.759 (0.131)^c^
COVID-19 cases per 1000 people	0.079 (0.020)^c^	0.128 (0.018)^c^	0.116 (0.016)^c^	0.090 (0.012)^c^	0.098 (0.011)^c^
Mean No. of COVID-19 deaths	4.50	4.59	3.77	3.56	3.14

Abbreviations: COVID-19, coronavirus disease 2019; ln, natural log (marginal effect of an increase of 1 unit of the natural log of the number of certified beds).

^a^ Marginal effects and SEs were determined using zero-inflated negative binomial regression analysis.

^b^ Nursing homes were categorized by racial composition quintile based on the percentage of White residents, with quintile 1 indicating 0% to 59.7%, quintile 2 indicating 59.8% to 81.0%, quintile 3 indicating 81.0% to 91.8%, quintile 4 indicating 91.8% to 97.3%, and quintile 5 indicating 97.3% to 100%.

^c^ *P* < .01.

^d^ *P* < .05.

In the secondary analyses, we examined whether these associations were different when nursing homes were classified as having high proportions of Black or Hispanic residents. The unadjusted numbers of cases and deaths were highest among nursing homes with the highest proportions of Black and Hispanic residents (eFigure 1 in the Supplement). In addition, as observed in the classification by percentage of White residents only, the differences were moderated by controlling for the number of certified beds and county-level cases but not by controlling for case mix or other nursing home characteristics (eFigure 2 in the Supplement). Results were also similar in an analysis using only confirmed cases (eFigure 3 in the Supplement) and cases among staff (eFigure 4 in the Supplement). The association between nursing home racial composition and deaths was consistent when examining data from earlier in the pandemic (May to June 2020) vs later in the pandemic (July to September 2020) (eTable 4, eTable 5, and eFigure 5 in the Supplement). The number of facility-level deaths per 100 cases decreased over time; however, the association between a higher proportion of White residents and a lower number of deaths per 100 cases was consistent in both of the periods examined (eTable 6 in the Supplement).

## Discussion

Our study examined the large differences in COVID-19 cases and deaths by the racial composition of nursing homes. Nursing homes in which more than 40% of residents were non-White experienced case and death counts that were 3.3-fold higher than those of nursing homes with low proportions of non-White residents. By sequentially adjusting for nursing home characteristics and county-level COVID-19 cases (ie, cases outside of nursing homes), we found that the differences in resident deaths by race were associated with nursing home size (as measured by the number of certified beds) and community-level outbreak severity. The aggregate health status of residents made little difference.

Two previous studies examined racial disparities among nursing home residents early in the COVID-19 pandemic. Using data from a limited number of states, 1 analysis found that early outbreaks in nursing homes occurred in facilities with the highest proportions of non-White residents.^18^ In a recent study, Li et al^3^ used national data similar to ours to examine 1 week in late May 2020 and reported differences by the racial composition of the facility’s residents. We not only examined differences in total resident deaths during the pandemic (to date) using more recent data on a larger scale, but we also identified possible explanations for these disparities.

The current pandemic has been described as a perfect storm^19^ for nursing home residents because of the worse health of this population and the realities of living in congregate settings that may have inadequate resources for testing and personal protective equipment in addition to staffing shortages. Although these factors have implications for all nursing homes, our findings suggest that facilities with higher proportions of non-White residents are experiencing worse outcomes than those with higher proportions of White residents. Researchers have consistently reported that segregation is present in nursing homes and that nursing homes with higher proportions of non-White residents are associated with worse-quality care.^7,15^ Our results suggest that the COVID-19 pandemic may have exacerbated this inequity. Factors associated with lower proportions of White nursing home residents have also been associated with lower nursing home quality in general and worse COVID-19 outcomes specifically. Because minority communities experience the highest rates of COVID-19 infection^1^ and nursing homes in those communities are generally of lower quality,^4,5,6,7,8^ non-White nursing home residents are in the eye of that perfect storm.

The negative consequences of the pandemic for nursing home residents are likely to be ameliorated, although not eliminated, with the emergence of vaccines and the prioritization of vaccine delivery to residents and staff in long-term care facilities. As vaccination proceeds, it will be important for policymakers to consider existing inequities to ensure that the process of vaccine distribution includes particular efforts to reach communities of color.

### Limitations

This study has several limitations. First, although facility-level characteristics are important, the current study was limited by the lack of individual-level data on COVID-19 cases and deaths among nursing home residents to study these disparities more explicitly. Individual-level data are needed to understand whether there are within-facility disparities in addition to the between-facility disparities identified in this study. More generally, future research using individual-level data on COVID-19 cases and deaths among nursing home residents is warranted to better understand the individual-level risk factors associated with worse COVID-19 outcomes, including mortality, among this disproportionally impacted population.

Second, our data did not allow for racial classifications other than White, Black, and Hispanic. More detailed data on race and ethnicity are needed to examine whether there are differences in COVID-19 outcomes among the diverse non-White groups. Third, COVID-19 case and death counts were self-reported, and nursing homes were only required to report these counts beginning in mid-May 2020, which omitted many previous cases and deaths from our analysis. Fourth, our case mix variables may not capture all relevant aspects of health. Fifth, this study was a cross-sectional observational analysis that may not represent causal associations.

## Conclusions

Consistent with historical racial disparities in long-term care and current pandemic-associated deaths, this study’s results suggest that nursing homes with higher proportions of non-White residents are experiencing the worst COVID-19 outcomes. Disparities in deaths by nursing home racial composition are associated with the disproportionately high spread of the disease in non-White communities and the characteristics of the nursing homes that serve those communities.

## Notes

Supplement. **eTable 1.** Sample Restrictions **eTable 2.** Full Results From Regression-Adjusted Models Presented in Figure 2 **eTable 3.** Marginal Effects From Negative Binomial Regression of COVID-19 Deaths **eTable 4.** Marginal Effects Estimates for Sequential Adjustment for Deaths Before June 29, 2020 **eTable 5.** Marginal Effects Estimates for Sequential Adjustment for Deaths Between June 29 and September 13, 2020 **eTable 6.** Unadjusted COVID-19 Deaths Per 100 Cases Among Nursing Home Residents by Nursing Home Racial Composition Quintile: Early and Late **eFigure 1.** Unadjusted COVID-19 Cases and Deaths Among Nursing Home Residents by Nursing Home Racial Composition Categories **eFigure 2.** Marginal Effects of Race Group on COVID-19 Deaths Among Nursing Home Residents **eFigure 3.** Unadjusted Confirmed COVID-19 Cases and Confirmed and Suspected COVID-19 Deaths Among Nursing Home Residents by Nursing Home Racial Composition Quintile **eFigure 4.** Unadjusted COVID-19 Cases Among Nursing Home Staff by Nursing Home Racial Composition Quintile **eFigure 5.** Unadjusted COVID-19 Cases and Deaths Among Nursing Home Residents by Nursing Home Racial Composition Quintile: Early and Late
