# Supplementary material for: Factors Associated With Racial Differences in Deaths Among Nursing Home Residents With COVID-19 Infection in the US
Source: JAMA Netw Open. 2021 Feb 10;4(2):e2037431. doi: 10.1001/jamanetworkopen.2020.37431 (PMC7876590; doi:10.1001/jamanetworkopen.2020.37431)
Supplement: Supplement. — eTable 1. Sample Restrictions eTable 2. Full Results From Regression-Adjusted Models Presented in Figure 2 eTable 3. Marginal Effects From Negative Binomial Regression of COVID-19 Deaths eTable 4. Marginal Effects Estimates for Sequential Adjustment for Deaths Before June 29, 2020 eTable 5. Marginal Effects Estimates for Sequential Adjustment for Deaths Between June 29 and September 13, 2020 eTable 6. Unadjusted COVID-19 Deaths Per 100 Cases Among Nursing Home Residents by Nursing Home Racial Composition Quintile: Early and Late eFigure 1. Unadjusted COVID-19 Cases and Deaths Among Nursing Home Residents by Nursing Home Racial Composition Categories eFigure 2. Marginal Effects of Race Group on COVID-19 Deaths Among Nursing Home Residents eFigure 3. Unadjusted Confirmed COVID-19 Cases and Confirmed and Suspected COVID-19 Deaths Among Nursing Home Residents by Nursing Home Racial Composition Quintile eFigure 4. Unadjusted COVID-19 Cases Among Nursing Home Staff by Nursing Home Racial Composition Quintile eFigure 5. Unadjusted COVID-19 Cases and Deaths Among Nursing Home Residents by Nursing Home Racial Composition Quintile: Early and Late [file jamanetwopen-e2037431-s001.pdf]

Supplementary Online Content

Gorges RJ, Konetzka RT. Factors associated with racial differences in deaths among nursing home residents with COVID-19 infection in the US. *JAMA Netw Open*. 2021;4(2):e2037431. doi:10.1001/jamanetworkopen.2020.37431

eTable 1. Sample Restrictions

eTable 2. Full Results From Regression-Adjusted Models Presented in Figure 2

eTable 3. Marginal Effects From Negative Binomial Regression of COVID-19 Deaths

eTable 4. Marginal Effects Estimates for Sequential Adjustment for Deaths Before June 29, 2020

eTable 5. Marginal Effects Estimates for Sequential Adjustment for Deaths Between June 29 and September 13, 2020

eTable 6. Unadjusted COVID-19 Deaths Per 100 Cases Among Nursing Home Residents by Nursing Home Racial Composition Quintile: Early and Late

eFigure 1. Unadjusted COVID-19 Cases and Deaths Among Nursing Home Residents by Nursing Home Racial Composition Categories

eFigure 2. Marginal Effects of Race Group on COVID-19 Deaths Among Nursing Home Residents

eFigure 3. Unadjusted Confirmed COVID-19 Cases and Confirmed and Suspected COVID-19 Deaths Among Nursing Home Residents by Nursing Home Racial Composition Quintile

eFigure 4. Unadjusted COVID-19 Cases Among Nursing Home Staff by Nursing Home Racial Composition Quintile

eFigure 5. Unadjusted COVID-19 Cases and Deaths Among Nursing Home Residents by Nursing Home Racial Composition Quintile: Early and Late

This supplementary material has been provided by the authors to give readers additional information about their work.

© 2021 Gorges RJ et al. *JAMA Network Open*.

eTable 1. Sample Restrictions

The following table describes sample restrictions required for the analyses. We began with all nursing homes certified by Medicare and Medicaid with entries in Nursing Home Compare. We then limited to facilities reporting to the CDC National Healthcare Safety Network COVID-19 Module for Long-Term Care Facilities at least one week with data meeting CMS data quality checks. Finally, we require a link at the facility-level to LTCfocus to determine facility share residents of white race and other nursing home characteristics that are used as controls in the adjusted models.

	N	% of all nursing homes
Nursing homes in Nursing Home Compare	15,439	
Reporting COVID data to CDC at least one week[1]	14,954	96.9
Link to LTCfocus	14,065	91.1
Not missing % white race variable[2]	14,063	91.1
Not missing other variables in models	13,312	86.2

Notes:

1. Reporting = Reporting data meeting CMS quality assurance check by the week ending September 13, 2020 in the CMS COVID-19 Nursing Home Dataset released December 4, 2020.
2. When Percent White Race = LNE in raw data, impute to Quintile 1 (low). LNE designation is due to LTCfocus not reporting due cell size less than 11 per CMS data reporting rules.

© 2021 Gorges RJ et al. *JAMA Network Open*.

eTable 2. Full Results From Regression-Adjusted Models Presented in Figure 2

Table 2 below reports the marginal effects from the sequentially adjusted models that are used to calculate the predicted means in Figure 2. Marginal effects for the resident percent white race quintiles are relative to Quintile 5 (highest share white race).

	COVID-19 Deaths	COVID-19 Deaths	COVID-19 Deaths	COVID-19 Deaths	COVID-19 Deaths
	(1)	(2)	(3)	(4)	(5)
% White Race Q1 (Low)	3.892	2.168	2.119	2.125	0.963
	(0.203)**	(0.192)**	(0.196)**	(0.203)**	(0.226)**
% White Race Q2	3.518	1.964	1.919	1.971	1.056
	(0.206)**	(0.189)**	(0.190)**	(0.193)**	(0.213)**
% White Race Q3	2.147	1.125	1.103	1.182	0.572
	(0.170)**	(0.176)**	(0.177)**	(0.177)**	(0.197)**
% White Race Q4	1.564	1.002	0.980	1.035	0.848
	(0.174)**	(0.186)**	(0.188)**	(0.188)**	(0.213)**
Ln(Certified beds)		5.192	5.131	5.048	4.831
		(0.166)**	(0.165)**	(0.165)**	(0.165)**
Case-Mix Index			0.034	0.037	0.019
			(0.049)	(0.049)	(0.049)
% Residents Hypertension			0.026	0.025	0.015
			(0.006)**	(0.006)**	(0.006)*
For-profit				0.003	0.040
				(0.170)	(0.168)
Government Owned				-0.704	-0.596
				(0.343)*	(0.339)
Chain				-0.307	-0.223
				(0.126)*	(0.125)
% Residents Medicaid				-0.007	-0.005
				(0.004)	(0.004)
% Residents Medicare				-0.006	-0.013
				(0.007)	(0.007)*
NHC Star Rating=1				-0.314	-0.338
				(0.201)	(0.200)
NHC Star Rating=2				0.147	0.170
				(0.197)	(0.195)
NHC Star Rating=3				-0.339	-0.363
				(0.204)	(0.202)
NHC Star Rating=4				-0.017	-0.027
				(0.182)	(0.180)
Adjusted Nursing Hours/Resident/Day				-0.456	-0.549
				(0.092)**	(0.091)**
COVID-19 cases/1,000 population (county)					0.105
					(0.007)**
N	13,312	13,312	13,312	13,312	13,312

* $p<0.05$; ** $p<0.01$

NHC=Nursing Home Compare. Star ratings are the overall star ratings.

Mean number of deaths = 3.9.

Marginal effects (standard errors) from zero-inflated negative binomial regression reported.

© 2021 Gorges RJ et al. *JAMA Network Open*.

eTable 3. Marginal Effects From Negative Binomial Regression of COVID-19 Deaths

	COVID-19 Deaths	COVID-19 Deaths	COVID-19 Deaths	COVID-19 Deaths	COVID-19 Deaths
% White Race Q1 (Low)	3.892	2.666	2.522	2.664	1.278
	(0.203)**	(0.206)**	(0.208)**	(0.224)**	(0.255)**
% White Race Q2	3.518	2.445	2.408	2.502	1.684
	(0.206)**	(0.200)**	(0.202)**	(0.212)**	(0.246)**
% White Race Q3	2.147	1.505	1.473	1.521	1.054
	(0.170)**	(0.188)**	(0.190)**	(0.190)**	(0.229)**
% White Race Q4	1.564	1.172	1.172	1.161	1.216
	(0.174)**	(0.191)**	(0.194)**	(0.190)**	(0.252)**
Ln(Certified beds)		6.007	5.921	5.931	6.085
		(0.243)**	(0.240)**	(0.248)**	(0.267)**
Case-Mix Index			0.176	0.165	0.116
			(0.061)**	(0.061)**	(0.065)
% Residents Hypertension			0.031	0.029	0.012
			(0.007)**	(0.007)**	(0.008)
For profit				-0.111	-0.005
				(0.210)	(0.224)
Government Owned				-1.309	-1.288
				(0.407)**	(0.442)**
Chain				-0.305	-0.145
				(0.153)*	(0.168)
% Residents Medicaid				-0.011	-0.011
				(0.005)*	(0.005)*
% Residents Medicare				0.004	-0.005
				(0.008)	(0.009)
NHC Star Rating=1				-0.200	-0.098
				(0.249)	(0.268)
NHC Star Rating=2				0.240	0.341
				(0.242)	(0.256)
NHC Star Rating=3				-0.326	-0.352
				(0.244)	(0.267)
NHC Star Rating=4				0.062	0.147
				(0.227)	(0.242)
Adjusted Nursing Hours/Resident /Day				-0.423	-0.617

© 2021 Gorges RJ et al. *JAMA Network Open*.

				(0.116)**	(0.130)**
COVID-19 cases/1,000 population (county)					0.161
					(0.010)**
N	13,312	13,312	13,312	13,312	13,312

* *p*<0.05; ** *p*<0.01
Mean number of deaths = 3.9.
Marginal effects (standard errors) from negative binomial regression reported.

We selected the zero-inflated negative binomial to model the count outcome of number of deaths per facility after examining the fit of several alternative models. The distribution of deaths has both a large number of facilities reporting zero deaths and over dispersion (i.e. a long right tail). Following Deb, Norton, and Manning (2017) we compared the fit of several alternative models by comparing the AIC and BIC and the zero-inflated negative binomial model had the best fit.

An alternative model is the negative binomial model, without the zero-inflated step. We repeated the sequential adjustment regression estimation with this alternative model and report the marginal effects estimates above in eTable 3. Results of the negative binomial model are consistent with the results we report in Figure 2 and eTable2 using the zero-inflated negative binomial model. Controlling for number of beds and county level COVID-19 cases per capita result in the largest decreases in the magnitudes of the marginal effects of each of the quintile groups relative to the nursing homes with the highest shares of white residents.

© 2021 Gorges RJ et al. *JAMA Network Open*.

eTable 4. Marginal Effects Estimates for Sequential Adjustment for Deaths Before June 29, 2020

	COVID-19 Deaths	COVID-19 Deaths	COVID-19 Deaths	COVID-19 Deaths	COVID-19 Deaths
	(1)	(2)	(3)	(4)	(5)
% White Race Quintile 1 (Low)	2.602	1.265	1.280	1.410	-0.099
	(0.182)**	(0.166)**	(0.168)**	(0.175)**	(0.191)
% White Race Quintile 2	2.141	1.005	1.008	1.137	0.073
	(0.171)**	(0.160)**	(0.161)**	(0.160)**	(0.183)
% White Race Quintile 3	1.294	0.588	0.598	0.689	-0.015
	(0.148)**	(0.158)**	(0.158)**	(0.154)**	(0.183)
% White Race Quintile 4	1.237	0.883	0.887	0.944	0.713
	(0.155)**	(0.171)**	(0.171)**	(0.166)**	(0.205)**
Ln (Certified beds)		4.043	4.027	3.957	3.120
		(0.148)**	(0.148)**	(0.147)**	(0.138)**
Case Mix Index			-0.083	-0.080	-0.045
			(0.043)	(0.043)	(0.045)
% Residents Hypertension			0.018	0.014	0.009
			(0.005)**	(0.005)**	(0.005)
For profit				-0.030	0.243
				(0.142)	(0.133)
Government Owned				-1.106	-0.498
				(0.303)**	(0.288)
Chain				-0.431	-0.034
				(0.107)**	(0.105)
% Residents Medicaid				-0.011	-0.006
				(0.003)**	(0.003)*
% Residents Medicare				-0.009	-0.017
				(0.006)	(0.005)**
Overall Star Rating=1				-0.659	-0.134
				(0.178)**	(0.172)
Overall Star Rating=2				-0.247	0.171
				(0.168)	(0.161)
Overall Star Rating=3				-0.645	-0.314
				(0.179)**	(0.172)
Overall Star Rating=4				-0.138	0.101
				(0.160)	(0.151)
Adjusted total nurse hours				-0.499	-0.339
				(0.084)**	(0.083)**
COVID-19 cases/1,000 population (county)					0.288
					(0.008)**
N	12,983	12,983	12,983	12,983	12,983

* $p<0.05$; ** $p<0.01$

Mean outcome = 2.56

Marginal effects from zero inflated negative binomial regression reported.

© 2021 Gorges RJ et al. *JAMA Network Open*.

eTable 5. Marginal Effects Estimates for Sequential Adjustment for Deaths Between June 29 and September 13, 2020

	COVID-19 Deaths	COVID-19 Deaths	COVID-19 Deaths	COVID-19 Deaths	COVID-19 Deaths
	(1)	(2)	(3)	(4)	(5)
% White Race Quintile 1 (Low)	1.212	0.861	0.779	0.596	0.003
	(0.088)**	(0.094)**	(0.097)**	(0.100)**	(0.117)
% White Race Quintile 2	1.293	0.932	0.895	0.753	0.286
	(0.108)**	(0.101)**	(0.103)**	(0.105)**	(0.123)*
% White Race Quintile 3	0.800	0.553	0.534	0.477	0.187
	(0.080)**	(0.087)**	(0.089)**	(0.096)**	(0.114)
% White Race Quintile 4	0.337	0.189	0.175	0.145	0.057
	(0.076)**	(0.085)*	(0.086)*	(0.094)	(0.122)
Ln(Certified beds)		1.114	1.083	1.066	1.166
		(0.088)**	(0.089)**	(0.086)**	(0.092)**
Case Mix Index			0.092	0.098	0.063
			(0.025)**	(0.025)**	(0.026)*
% Residents Hypertension			0.011	0.012	0.002
			(0.003)**	(0.003)**	(0.003)
For profit				0.110	0.053
				(0.086)	(0.087)
Government Owned				0.279	0.233
				(0.172)	(0.188)
Chain				0.232	0.149
				(0.067)**	(0.067)*
% Residents Medicaid				0.006	0.004
				(0.002)**	(0.002)
% Residents Medicare				0.001	-0.003
				(0.003)	(0.003)
Overall Star Rating=1				0.333	0.114
				(0.103)**	(0.103)
Overall Star Rating=2				0.385	0.271
				(0.102)**	(0.102)**
Overall Star Rating=3				0.265	0.101
				(0.106)*	(0.106)
Overall Star Rating=4				0.122	0.023
				(0.096)	(0.094)
Adjusted total nurse hours				0.128	-0.079
				(0.045)**	(0.045)
COVID-19 cases/1,000 population (county)					0.095
					(0.004)**
N	13,093	13,093	13,093	13,093	13,093

* $p<0.05$; ** $p<0.01$

Mean outcome = 1.27

Marginal effects from zero inflated negative binomial regression reported.

As in the main analyses, the effects of race were moderated as we added both certified beds and county-level cases outside of the nursing home to the model when modeling deaths that occurred both earlier and later in the pandemic.

© 2021 Gorges RJ et al. *JAMA Network Open*.

Interestingly, NHC star rating was not associated with the number of deaths prior to July; however, star ratings of 1 and 2 were associated with more deaths later in the pandemic, during July-September.

© 2021 Gorges RJ et al. *JAMA Network Open*.

eTable 6. Unadjusted COVID-19 Deaths Per 100 Cases Among Nursing Home Residents by Nursing Home Racial Composition Quintile: Early and Late

Quintile % White Race (Min-Max)	1 (low) (0.0 - 59.7)	2 (59.8 - 81.0)	3 (81.0 - 91.8)	4 (91.8 - 97.3)	5 (high) (97.3 - 100.0)
Panel a: Early period, through 6/28/2020					
Deaths per 100 Cases, Mean (SD)	16.4 (25.1)	16.2 (25.3)	13.5 (23.2)	12.6 (22.9)	7.8 (18.1)
N	2,523	2,570	2,626	2,647	2,617
Panel b: Late period, 6/29/2020 – 9/13/2020					
Deaths per 100 Cases, Mean (SD)	12.4 (23.2)	13.4 (24.9)	10.7 (22.3)	8.5 (20.0)	6.0 (16.4)
N	2,550	2,618	2,657	2,654	2,636

While it appears that deaths per 100 cases have declined over time, during both periods nursing homes with a higher share of non-white residents experienced more deaths per 100 reported cases. This is consistent with the overall trend in deaths/100 cases we report in Table 1 as well as our findings focusing on the raw count of deaths.

© 2021 Gorges RJ et al. *JAMA Network Open*.

eFigure 1. Unadjusted COVID-19 Cases and Deaths Among Nursing Home Residents by Nursing Home Racial Composition Categories

We explore alternative categorizations of the race composition of nursing home residents using the percent Black and percent Hispanic variables from LTCfocus. We divide the sample of nursing homes into five groups (1) High share (>95%) White; (2) Low shares White, Black, and Hispanic; (3) High share Black (>15%) and low share Hispanic; (4) High share Hispanic (>5%) and low share Black; (5) High share Black (>15%) and Hispanic (>5%). We repeat the main figures using these alternative classifications.

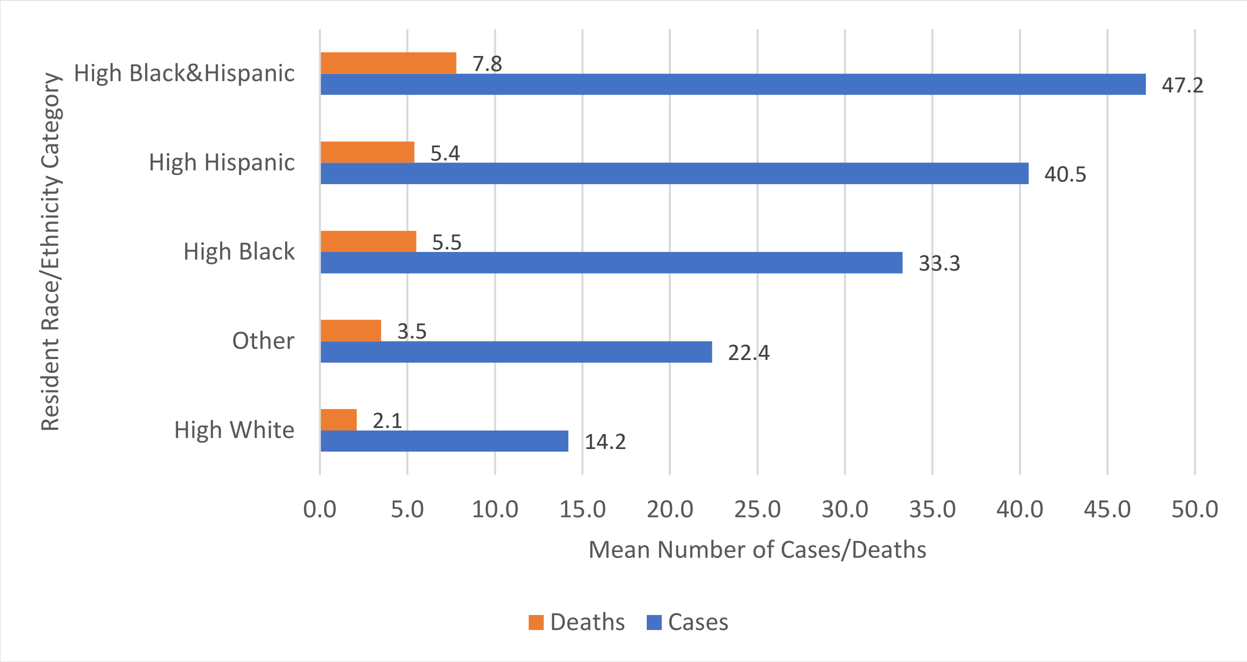

Source: CMS COVID-19 Nursing Home dataset released December 4, 2020 merged with LTCfocus and Nursing Home Compare.
Limited to facilities reporting COVID-19 case data meeting quality assurance checks and non-missing resident % race (n=13,312).
Cases (deaths) are total confirmed and suspected COVID-19 cases (deaths) among residents, through week ending September 13, 2020.
Quintile cutoffs for percent residents' race are: High White>95%; High Black>15%; and High Hispanic>5%.

© 2021 Gorges RJ et al. *JAMA Network Open*.

eFigure 2. Marginal Effects of Race Group on COVID-19 Deaths Among Nursing Home Residents

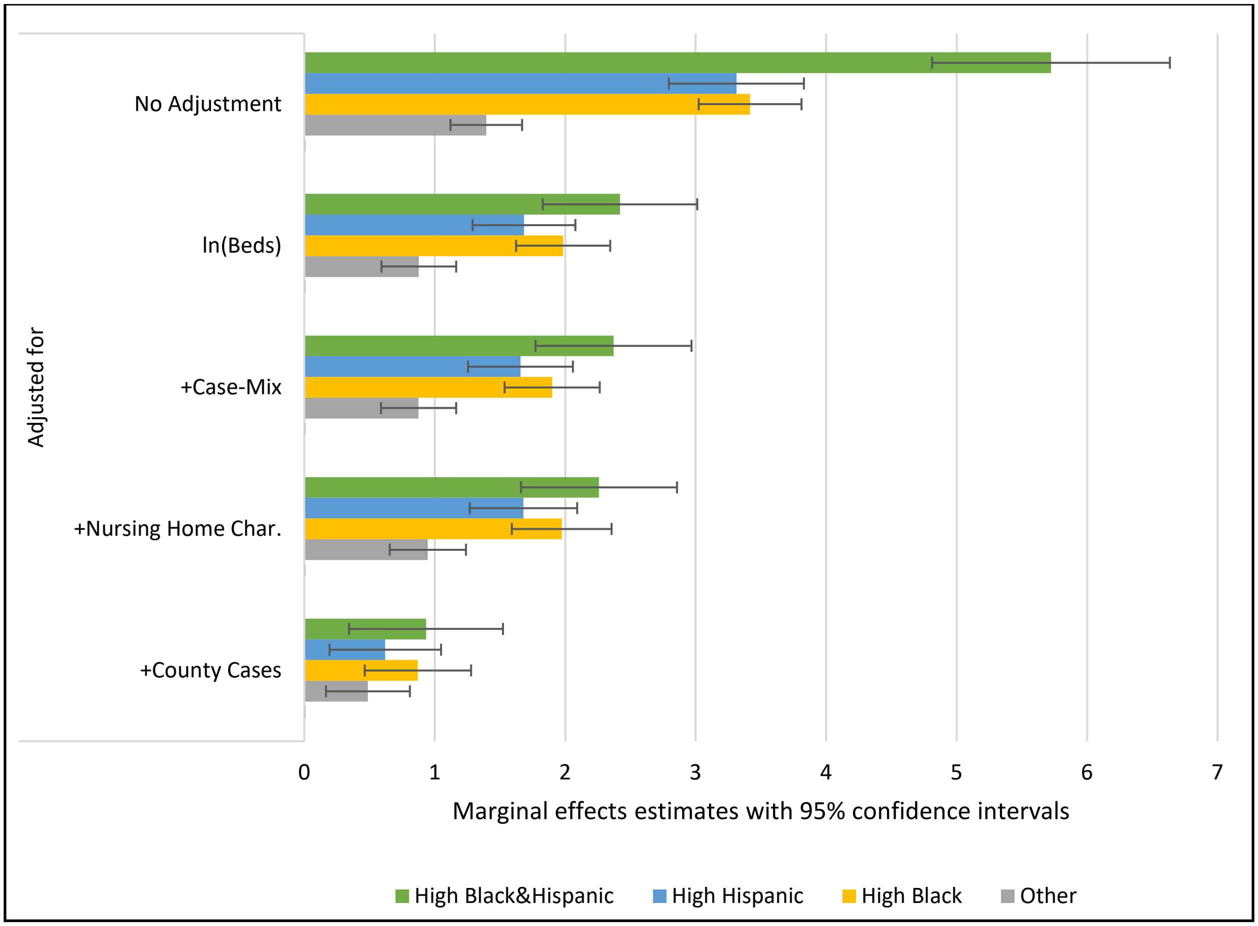

Notes: Marginal effects and 95% confidence intervals from zero-inflated negative binomial regression. Marginal effects are relative to the high White group (nursing homes with >95% White residents). Model labeled "No Adjustment" calculates differences from the high White group in mean death counts. Ln(beds) adjusts for number of certified beds only. +Case-mix is adjusted for number of beds, acuity index and percent residents with hypertension. + Nursing home characteristics is adjusted for number of beds, case mix and characteristics in Table 1 (ownership, chain, % residents Medicaid-paid, % residents Medicare-paid, Nursing Home Compare overall star rating, and adjusted nursing hours/resident/day). Finally, + County Cases is adjusted for county confirmed COVID-19 cases (excluding nursing home resident cases) per 1,000 population in the county using quadratic functional form ($x + x^2$) in addition to number of beds, case-mix, and nursing home characteristics.

© 2021 Gorges RJ et al. *JAMA Network Open*.

eFigure 3. Unadjusted Confirmed COVID-19 Cases and Confirmed and Suspected COVID-19 Deaths Among Nursing Home Residents by Nursing Home Racial Composition Quintile

CMS separately repots confirmed and suspected COVID-19 cases among residents. To examine whether the association between total confirmed and suspected cases and race composition in Figure 1 was driven by the inclusion of suspected cases, we repeated the tabulation of number of cases by race quintile limiting to only confirmed cases in eFigure 3 below.

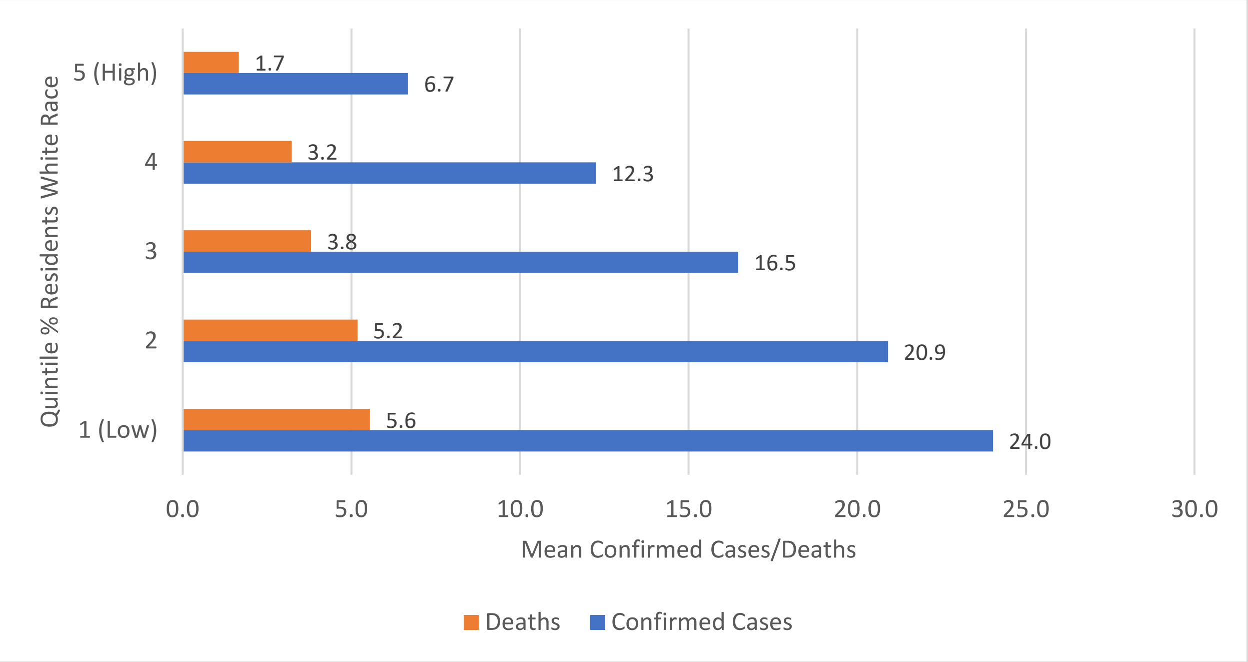

Source: CMS COVID-19 Nursing Home dataset released December 4, 2020 merged with LTCfocus and Nursing Home Compare.
Limited to facilities reporting COVID-19 case data meeting quality assurance checks and non-missing resident % race (n=13,312).
Cases are confirmed COVID-19 cases and deaths are total confirmed and suspected COVID-19 deaths among residents, through week ending September 13, 2020.
Quintile cutoffs for percent residents white race are 1 (Low)= 0-60%; 2=60-81%; 3=81-92%; 4=92-97%; 5 (High) 97-100%.

© 2021 Gorges RJ et al. *JAMA Network Open*.

eFigure 4. Unadjusted COVID-19 Cases Among Nursing Home Staff by Nursing Home Racial Composition Quintile

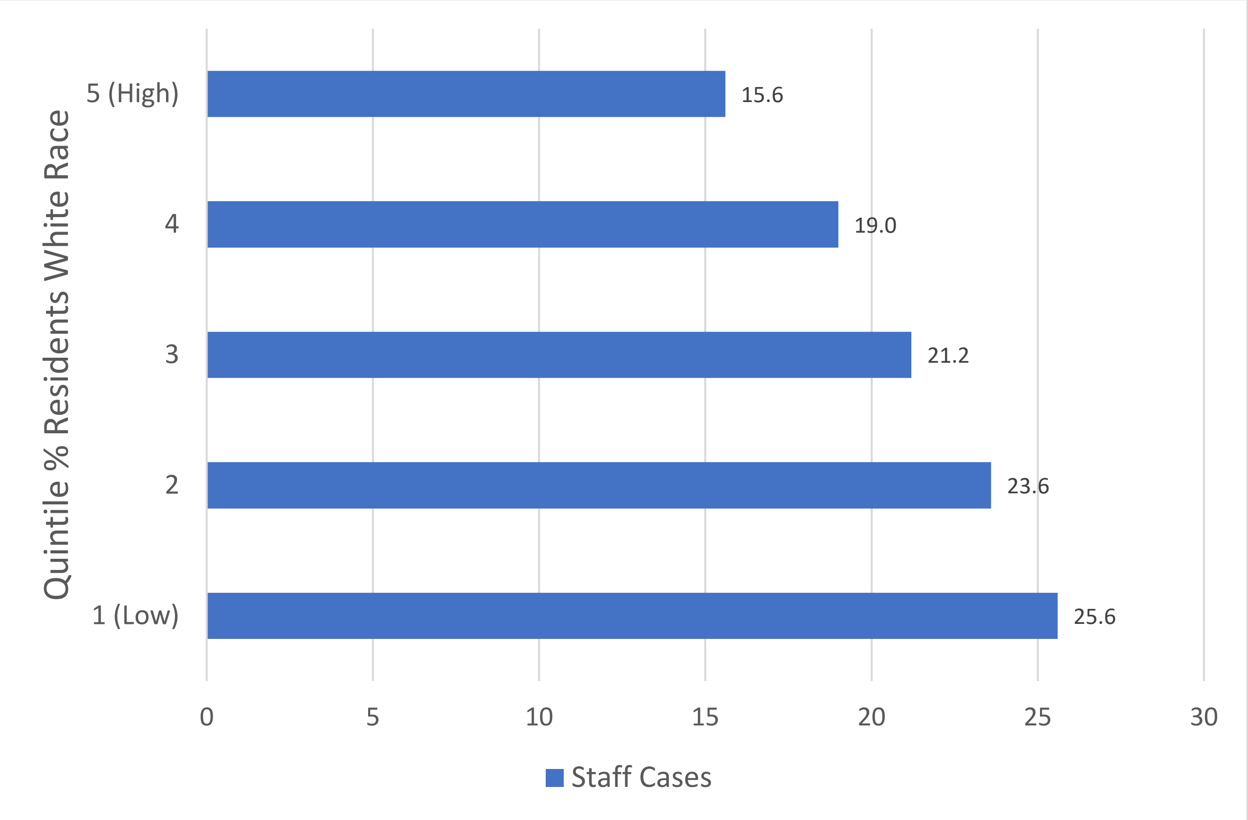

Source: CMS COVID-19 Nursing Home dataset released December 4, 2020 merged with LTCfocus and Nursing Home Compare.
Limited to facilities reporting COVID-19 case data meeting quality assurance checks and non-missing resident % race (n=13,312).
Cases are confirmed and suspected COVID-19 cases among staff, through week ending September 13, 2020.
Quintile cutoffs for percent residents white race are 1 (Low)= 0-60%; 2=60-81%; 3=81-92%; 4=92-97%; 5 (High) 97-100%.

By mid-September, nearly all facilities had experienced at least one suspected or confirmed COVID-19 case among staff. However, the association between more COVID-19 cases and higher share of non-white residents is present among staff; the mean number of staff cases increases monotonically with the quintile % residents white race.

© 2021 Gorges RJ et al. *JAMA Network Open*.

eFigure 5. Unadjusted COVID-19 Cases and Deaths Among Nursing Home Residents by Nursing Home Racial Composition Quintile: Early and Late

Finally, we explore whether relationships between race, county COVID-19 community cases, case-mix, and nursing home characteristics have changed over time during the pandemic. To do this, we divided the total cases/deaths into cases/deaths that occurred prior to June 29 to capture cases/deaths "early" in the pandemic and cases/deaths that occurred between June 29-September 13 to capture cases/deaths "late" in the pandemic. We then repeated the main analyses separately for the early and late outcomes.

eFigure 5 below describes the relationship between nursing home race composition and the number of cases and deaths with the first panel illustrating the relationship early in the pandemic (through June) and the second panel showing it later in the pandemic (July-mid September). In each time period, there is a strong relationship between the share of residents of white race and cases/deaths, just as in the main results. The unadjusted pattern, whereby nursing homes serving more non-white residents experience case and death counts at much higher rates than homes with few non-white residents, does not appear to have changed over time.

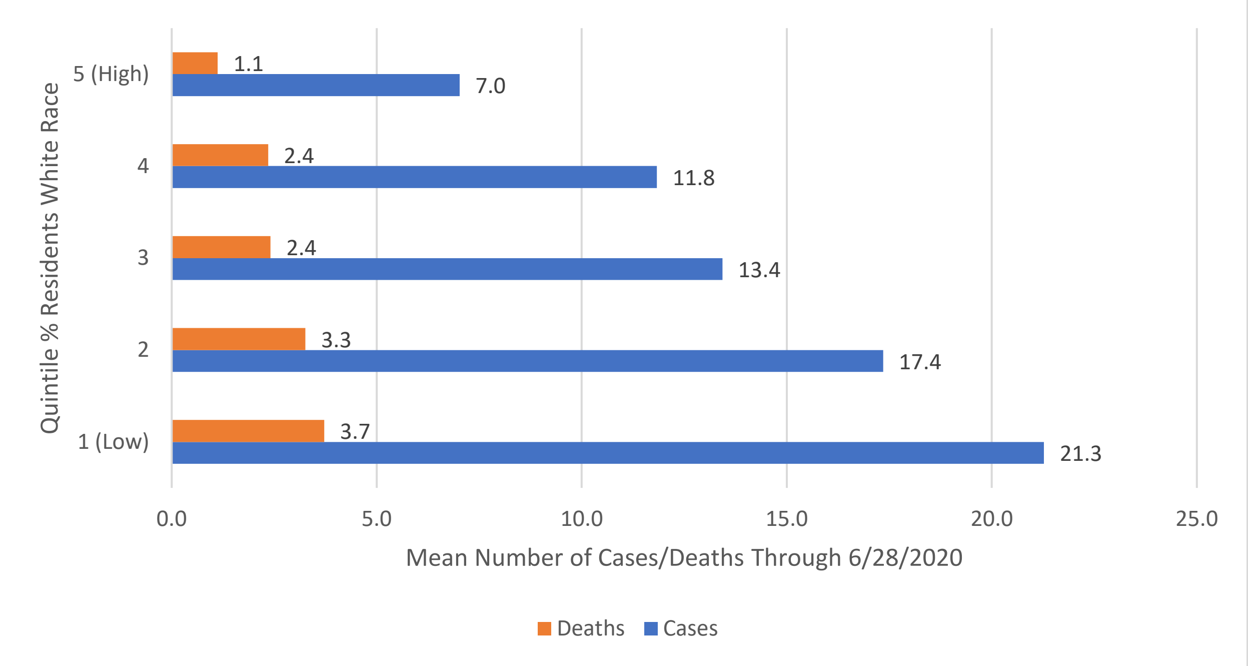

eFigure 5(a) Early Cases/Deaths

© 2021 Gorges RJ et al. *JAMA Network Open*.

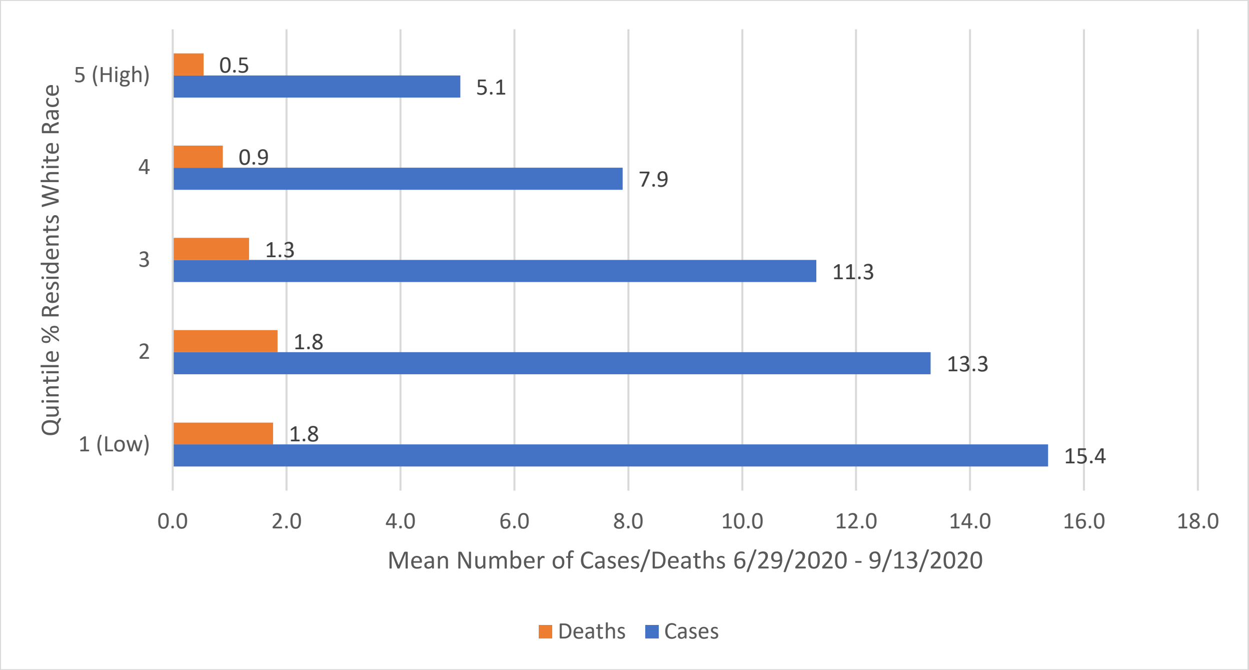

eFigure 5(b) Late Cases/Deaths

In each time period, there is a strong relationship between the share of residents of white race and cases/deaths, just as in the main results. The unadjusted pattern, whereby nursing homes serving more non-white residents experience case and death counts at much higher rates than homes with few non-white residents, does not appear to have changed over time.

© 2021 Gorges RJ et al. *JAMA Network Open*.

© 2021 Gorges RJ et al. *JAMA Network Open*.
